# The Promise and Challenge of Therapeutic MicroRNA Silencing in Diabetes and Metabolic Diseases

**DOI:** 10.1007/s11892-016-0745-3

**Published:** 2016-04-25

**Authors:** Praveen Sethupathy

**Affiliations:** Department of Genetics, School of Medicine, UNC Chapel Hill, Chapel Hill, NC 27599 USA; Lineberger Comprehensive Cancer Center, School of Medicine, UNC Chapel Hill, Chapel Hill, NC 27599 USA

**Keywords:** MicroRNA, Therapeutics, Anti-sense oligonucleotide, Diabetes, Metabolic disease, Aptamer

## Abstract

MicroRNAs (miRNAs) are small, non-coding, RNA molecules that regulate gene expression. They have a long evolutionary history and are found in plants, viruses, and animals. Although initially discovered in 1993 in *Caenorhabditis elegans*, they were not appreciated as widespread and abundant gene regulators until the early 2000s. Studies in the last decade have found that miRNAs confer phenotypic robustness in the face of environmental perturbation, may serve as diagnostic and prognostic indicators of disease, underlie the pathobiology of a wide array of complex disorders, and represent compelling therapeutic targets. Pre-clinical studies in animal models have demonstrated that pharmacologic manipulation of miRNAs, mostly in the liver, can modulate metabolic phenotypes and even reverse the course of insulin resistance and diabetes. There is cautious optimism in the field about miRNA-based therapies for diabetes, several of which are already in various stages of clinical trials. This review will highlight both the promise and the most pressing challenges of therapeutic miRNA silencing in diabetes and related conditions.

## Introduction

Recent advances in sequencing technology have unveiled the diversity and complexity of both small and long non-coding RNAs (ncRNAs). Small RNAs, including short interfering RNAs (siRNAs) and microRNAs (miRNAs), mediate post-transcriptional gene silencing by RNA interference (RNAi), which was designated as the “Breakthrough of the Year” by the journal *Science* in 2002 and was the discovery for which the Nobel Prize in Physiology or Medicine was awarded in 2006. Furthermore, also in 2006, two specific miRNAs were selected as “Molecule of the Year” by the International Society for Molecular and Cell Biology and Biotechnology Protocols and Researchers (ISMCBBPR) based on the discovery that their tissue expression levels can accurately predict lung cancer prognosis. Since then, miRNAs have emerged as candidate circulating biomarkers of physiologic status [1]; potent regulators of diverse biological pathways from differentiation [2] to energy metabolism [3]; adaptive responders to environmental stimuli such as dietary nutrients [4], microbes [5], and pollutants [6]; inter-organ messengers in health and disease [7•]; and potential therapeutic targets in a wide array of disorders [8]. One context in which the role of miRNAs is increasingly appreciated is type 2 diabetes (T2D) [9], as well as related metabolic conditions such as obesity and hyperlipidemia.

The discovery of the importance of miRNAs to the etiology of these diseases was greatly facilitated by the advent of anti-sense technology for the study of miRNAs in vivo [10]. Anti-miRNA oligonucleotides (AMOs), also referred to as antagomiRs, are chemically modified stretches of nucleic acids that are partially or wholly complementary to specific target miRNAs. AMOs bind their target miRNAs with high specificity and potently suppress their molecular functions. In order to be effective in vivo, AMOs must be stable in circulation and readily taken up by tissues, which are features that are conferred by several different types of chemical modifications. The different AMO chemistries have been summarized previously [11]. One popular modification is the locked nucleic acid (LNA), wherein one or more monomers along the length of an AMO are locked in a specific conformation that enhances base stacking and increases the stability of base pairing with its target miRNA by 2–8 °C per locked monomer. The locked conformation is also refractory to digestion by exo- and endo-nucleases, thereby increasing the stability of AMOs in circulation. AMOs are increasingly used for in vivo research studies of miRNA function and are being developed for potential therapeutic use as well [12]. For example, LNA-mediated inhibition of miR-122 (initially tested in non-human primate models [13]) in patients with chronic hepatitis C was shown in phase 2a clinical trials to be non-immunogenic and highly effective at reversing the course of the disease by promoting viral clearance [14]. The success of LNA-anti-miR-122 (miravirsen) has generated sustained interest in the use of AMOs for in vivo silencing of additional miRNAs in other health conditions, most notably cancers and metabolic diseases.

## Promising Pre-clinical Examples of Therapeutic MicroRNA Silencing in Metabolic Disease

Numerous studies in the last few years have used AMOs for therapeutic silencing of at least ten different miRNAs in animal models to study their roles in diabetes, as well as related conditions including obesity, hyperlipidemia, and insulin resistance, and their potential as therapeutic targets (Table 1).

**Table 1:** Pre-clinical animal studies of anti-miRNA oligonucleotide (AMO) based therapeutics in diabetes and related metabolic conditions

MicroRNA	Metabolic phenotype/disease	References
let-7	Type 2 diabetes	Frost and Olson, PNAS [15]
miR-34	Obesity/type 2 diabetes	Fu et al., PNAS [16] Choi et al., Aging Cell [17]
miR-103/107	Obesity/type 2 diabetes	Trajkovski et al., Nature [18••]
miR-802	Obesity/type 2 diabetes	Kornfeld et al., Nature [19]
miR-208a	Obesity/type 2 diabetes	Grueter et al., Cell [20]
miR-181a	Insulin resistance/type 2 diabetes	Zhou et al., Diabetologia [21]
miR-379	Obesity/hyperlipidemia	deGuia et al., EMBO Journal [22]
miR-146b	Obesity	Ahn et al., EMBO Molecular Medicine [23]
miR-24	Steatosis/hyperlipidemia	Ng et al., Hepatology [24]
miR-29	Hyperlipidemia	Kurtz et al., Scientific Reports [25]

Below are highlighted specific examples in obesity/T2D, as well as hyperlipidemia.

### Obesity and Type 2 Diabetes

Obesity and T2D are complex metabolic disorders that affect hundreds of millions of people worldwide, have no cure, and are associated with numerous debilitating co-morbidities. At the physiologic level, T2D is characterized by resistance to insulin action in peripheral tissues such as the liver, adipose, and muscle and/or failure of pancreatic beta cells to produce/secrete enough insulin to meet metabolic demands, resulting in chronically elevated blood glucose levels (hyperglycemia) and numerous downstream complications. Both obesity and T2D are growing epidemics and critically require new and effective preventative and therapeutic strategies.

In 2011, Trajkovski et al. [18••] published a seminal study on therapeutic silencing of miRNAs in a mouse model of obesity/T2D. Specifically, they administered 2′-O-methyl modified AMOs by tail-vein injection to silence both miR-103 and miR-107 in the livers and adipose of *ob/ob* mice as well as wild-type C57BL/6J mice on high-fat diet (HFD) for 12 weeks. The effects of the AMO on miR-103/107 levels in other tissues were not reported. They found in both animal models that AMO-mediated suppression of miR-103 and miR-107, which are elevated in the livers of rodents and humans with insulin resistance, significantly alleviate hyperglycemia in large part by promoting insulin signaling in the liver and adipose. Specifically, while glucose uptake by muscle was not affected, glucose production and glycogen content in the liver, as well as adipocyte size, were significantly reduced. Overall, the findings from this study revealed that both miR-103 and miR-107 are key negative regulators of insulin signaling and also suggested that they are novel candidate therapeutic targets for T2D. More recent studies have shown that miR-107 levels may be regulated by inflammatory cues [26] in addition to dietary lipids [27] and that miR-107 is involved in the control of circadian rhythm [27], indicating that both miR-103 and miR-107 likely have broader roles in T2D etiology than through regulation of insulin signaling alone. In April 2015, Regulus Therapeutics announced that a GalNAc-conjugated AMO against miR-103/107 (RG-125) was selected by AstraZeneca for clinical development for the treatment of hepatic steatosis in patients with T2D and/or insulin resistance (http://ir.regulusrx.com/releasedetail.cfm?ReleaseID=905305).

In 2013, Choi et al. [17] used 2′-O-methoxyethyl phosphorothioate-modified AMOs to silence expression and activity of miR-34a (AMO34) in a mouse model of obesity (8–12-week-old C57BL6/J mice fed HFD for 20 weeks). AMO34a treatment was carried out by tail-vein injection three times over a 12-day period. Hepatic miR-34a levels, which were aberrantly elevated in the chronic HFD-fed mice before dosing, were rescued to levels seen in gender- and age-matched lean mice after the full course of AMO34a treatment. The effects of AMO34a on miR-34a levels in other tissues were not reported. Notably, AMO34a treatment led to significant improvements in glucose tolerance and insulin sensitivity, matching levels observed in lean mice. Furthermore, silencing of miR-34a also significantly reduced serum levels of IL-6, insulin, and triglycerides, although levels of alanine transaminase (ALT) and aspartate transaminase (AST) were unaffected. These findings indicate that AMO34a treatment can correct the metabolic imbalance induced by chronic HFD without side effects of liver inflammation or other toxicities. A subsequent study in 2014 from the same group showed that suppression of miR-34a in adipose tissue reduces adiposity and improves metabolic phenotypes in diet-induced obese mice by promoting beige and brown fat formation [28]; however, these experiments were conducted with lentiviral inhibition constructs since AMOs are not as efficiently taken up by adipose compared to the liver.

Also in 2013, Kornfeld et al. [19] showed that miR-802 is significantly increased in the liver of two different mouse models of obesity and insulin resistance: (1) 8–12-week-old mice homozygous for a diabetes mutation in the leptin receptor gene (*db/db*) and (2) 8–12-week-old mice fed HFD. They also demonstrated that hepatic miR-802 levels are significantly correlated with body mass index (BMI) in human subjects. These findings suggest that miR-802 may be linked to the development of systemic insulin resistance. Kornfeld et al. intravenously delivered small LNAs against miR-802 to HFD-fed mice on two consecutive days. Two weeks after the final dose, the authors observed remarkable suppression of miR-802 in the liver and kidney, but not in any other tissue. The inhibition of miR-802 led to a significant improvement in glucose tolerance, and this effect was not mediated by increased glucose uptake by the skeletal muscle or white adipose tissue, but rather enhanced insulin sensitivity in the liver (as measured by hepatic glucose production). miR-802 is relatively new on the miRNA scene, and much more remains to be uncovered about its functions in the liver as well as in other tissues where it is robustly expressed. For example, the effects of LNA-mediated suppression of miR-802 in the kidney have not yet been investigated. However, there is reason for cautious optimism regarding miR-802 as a compelling therapeutic target for obesity and insulin resistance.

### Hyperlipidemia and Type 2 Diabetes

Hyperlipidemia refers to the chronic elevation of lipids (cholesterol and fat) in the bloodstream, which is a significant risk factor for a variety of metabolic diseases, including T2D. The condition is most commonly managed by modification of diet, as well as drugs such as statins and fibrates. However, these medications are not uniformly effective across individuals in the human population, and in some cases, long-term and/or high-dose usage could even lead to very harmful side effects such as severe muscle damage [29], which may not be curable if not detected and treated sufficiently early. Therefore, there remains a critical need for new, safe, and effective medications to combat hyperlipidemia. Anti-gene inhibitors are already in various stages of clinical testing for hyperlipidemia. For example, treatment with an anti-sense oligonucleotide inhibitor of the gene apolipoprotein C-III (*APOC3*), a critical regulator of plasma triglyceride levels, led to safe, dose-dependent, and long-term reductions in plasma triglycerides in phase II clinical trials [30].

In a recent study, Kurtz et al. [25] administered LNAs by tail-vein injection in 8-week-old wild-type C57BL6/J mice to inhibit miR-29 family members (LNA29) in vivo. One week post-dosing, the LNAs were most effectively taken up by the liver and kidney, and as in previous studies, the LNAs did not successfully cross the blood–brain barrier. Treatment with the LNA29 significantly reduced plasma triglyceride levels by ∼15 % and cholesterol levels by ∼40 %, which is commensurate with the effects of statins, the most widely prescribed class of medications for hypercholesterolemia. The authors further demonstrated that the striking phenotypic effects of LNA29 were due in large part to suppression of lipogenic pathways in the liver. These findings raise the possibility that LNA29 could be used to lower cholesterol levels in diet-based or genetic animal models of hypercholesterolemia. Importantly, miR-29 has also been implicated in the control of insulin signaling [31], and therefore, inhibition of miR-29 may improve hepatic or even systemic insulin sensitivity as well, though this has not yet been demonstrated. Indeed, hepatic miR-29 is significantly elevated in a rat genetic model of diabetes and restored to normal levels upon treatment with Pioglitazone [32]. However, it should also be noted that miR-29 has also been shown to regulate a variety of other biological processes, including fibrosis [33, 34], immune response [35], and cell proliferation [36]. Therefore, future studies will need to assess comprehensively the systemic influences of AMO-mediated suppression of miR-29 and whether any potential side effects can be mitigated or circumvented with alternate dosing schemes or cocktail therapies.

In another study, Ng et al. [24] injected LNAs against miR-24 (LNA24) weekly for 4 weeks into 16-week-old wild-type C57BL6/J mice that were fed a HFD for 8 weeks. After completion of the dosing scheme, miR-24 levels were dramatically reduced in the liver, although the efficacy of inhibition in other tissues was not reported. While the LNA24 treatment had no effect on the overall body or liver weight, it significantly reduced fat accumulation in both the liver and the plasma of the HFD-fed mice. The authors further demonstrated that the effect of LNA24 was mediated by increased expression and activity of the insulin induced gene 1 (Insig1) protein, which is responsible for sequestering in the endoplasmic reticulum the key transcription factor responsible for lipid production in the liver, sterol regulatory element binding protein 1 (Srebp1) [37]. Therefore, silencing of miR-24, as in the case of silencing of miR-29, leads to the suppression of hepatic lipogenesis, suggesting that LNA24 may be a therapeutic strategy for metabolic conditions in which lipid levels are pathologic. Recently, miR-24 was also identified as a critical regulator of pancreatic beta cell function [38], in part through direct regulation of *Hnf1a* and *Neurod1*, which underlie the etiology of maturity-onset diabetes of the young (MODY). Furthermore, it was shown that suppression of aberrantly elevated miR-24 in pancreatic islets from HFD-fed mice significantly improves glucose-stimulated insulin secretion. These findings suggest that miR-24-based therapies may be relevant for T2D as well. Further studies are required to evaluate the effects of long-term dosing with LNA24, as well as to assess the efficacy of treatment in more severe metabolic disease models (e.g., long-term HFD).

### Other Conditions of Metabolic Dysfunction

Some cardio-metabolic diseases quite different from T2D still share some features of metabolic dysfunction. One such disorder of growing public health concern is atherosclerosis, which is characterized by the buildup of arterial plaque (cholesterol, triglycerides, calcium, and other cellular debris) that causes the hardening and constriction of arteries, leading to vascular disease, coronary heart disease, stroke, and death. It is the most prevalent disease in the developed world and is predicted to be the number one cause of death worldwide by the year 2020. Some medications, including those that lower plasma total cholesterol levels and improve lipoprotein profiles, can be used for treatment. However, the development of new, complementary therapeutic approaches for the regression of atherosclerosis remains an open and important area of biomedical investigation. The first and best-studied miRNA in the context of atherosclerosis is miR-33, which has two isoforms, miR-33a and miR-33b, encoded within the genes sterol regulatory element binding factor 2 (*Srebf2*) and sterol regulatory element binding factor 1 (*Srebf1*), respectively. The proteins encoded by these two genes are master transcriptional regulators of cholesterol and fatty acid synthesis [37]. In 2010, several independent studies used AMOs against miR-33 (AMO33) in mice to establish an important functional role for miR-33 in the maintenance of systemic cholesterol homeostasis [39–41]. Follow-up studies in mouse models of atherosclerosis (e.g., *Ldlr*^*−/−*^) produced conflicting reports about the safety and efficacy of long-term treatment [42, 43]. However, in vivo studies in non-human primates appear to suggest that therapeutic silencing of miR-33 is safe and atheroprotective, both in the short and long term [44, 45]. The latter findings indicate that AMO33 could remain a candidate therapeutic strategy for atherosclerosis.

## Key Challenges in Therapeutic MicroRNA Silencing

Despite the exciting and rapid pace of advance in the development and pre-clinical use of potent miRNA silencing technologies, there remain several major challenges for clinical development, three of which I will highlight here.

1. The effects of long-term (>5 weeks) AMO-mediated silencing of miRNAs are simply not known in most cases and require more thorough investigation. One notable exception is miR-33. While short-term (<5 weeks) silencing of miR-33 in mice was shown to be highly safe and efficacious by multiple groups, the data for long-term silencing has been conflicting. Although studies demonstrated that long-term silencing of miR-33 is safe in non-human primates, the mouse results provide caution enough to researchers that the effects of prolonged AMO treatment must be evaluated rigorously and comprehensively before proceeding to clinical trials.
2. The use of larger animal models for pre-clinical studies is critical but still limited. While rodent-based models are among the most widely used, larger animals such as pigs and monkeys are more physiologically relevant from a therapeutic standpoint. Also, the extent to which rodent miRNA networks are conserved in humans is still an open question, and it is important to consider pre-clinical studies in animals that are less evolutionarily distant from humans. Rodent models are incredibly valuable, in part because of the availability of rich resources, and they will remain an important part of the fabric of translational science. However, it will be important to develop more resources for studies of larger animal models in order to gain confidence in the relevance of a particular AMO for potential clinical trials.
3. AMO-based therapeutic silencing of miRNAs is currently heavily biased toward the liver (Table 1), in large part because the liver (as well as the kidney) is most effective at taking up circulating AMOs. While liver miRNAs contribute considerably to the governance of metabolic phenotypes, miRNAs in other tissues likely also have critical roles in the development of metabolic disease, such as miR-200a, which regulates pancreatic beta cell survival in type 2 diabetes [46•]; miR-375, which modulates insulin secretion from beta cells [47] and maintains alpha/beta cell mass in pancreatic islets [48]; and miR-7a, which controls the physiologic adaptation of beta cells to the metabolic conditions of obesity and type 2 diabetes [49]. Pancreatic islets and most other metabolic tissues such as adipose and skeletal muscle do not efficiently take up the type of AMOs currently in use. Alternate strategies are clearly needed in order to perform therapeutic silencing of miRNAs in tissues other than the liver and kidney.

## Conclusions and Future Outlook of Therapeutic MicroRNA Silencing

A few different approaches have been suggested in the literature to expand therapeutic silencing of miRNAs beyond the liver. One strategy in particular holds substantial promise: aptamer-AMO chimeras [50, 51••]. Specifically, an AMO can be chemically linked to an RNA aptamer, which is a structured RNA molecule designed to have high-affinity binding to a cell-surface receptor that is specific to a particular cell type. Once the aptamer-AMO chimera binds to the target receptor and is internalized, the link between the aptamer and the AMO is severed by Dicer, releasing the AMO in the cytoplasm. In this approach, the RNA aptamer essentially serves as a “guide” for the AMO to specific cell types. RNA aptamers can be selected for specific receptors of interest by high-throughput screening of large RNA libraries using strategies such as Systematic Evolution of Ligands by Exponential Enrichment (SELEX) [52]. Aptamer-siRNA chimeras have already been used effectively in xenografted mice to target particular oncogenes for knockdown only in cancer cells (using an aptamer designed for EpCAM, a cell surface antigen that is highly expressed in most epithelial cancers) in order to suppress tumor growth [53]. CD4 aptamer-siRNA chimeras have also been used to inhibit the transmission of human immunodeficiency virus (HIV) in humanized mice [54]. Aptamer-AMO chimeras represent the next frontier for cell-type specific therapeutic silencing of miRNAs in metabolic disorders, as well as a wider array of diseases.
